# Smart Roadside System for Driver Assistance and Safety Warnings: Framework and Applications

**DOI:** 10.3390/s110807420

**Published:** 2011-07-25

**Authors:** Jeong Ah Jang, Hyun Suk Kim, Han Byeog Cho

**Affiliations:** Vehicle-Ship IT Convergence Research Department, ETRI (Electronics and Telecommunications Research Institute), 161 Gajeong-dong, Yuseong-gu, Daejeon 305-350, Korea; E-Mails: hyskim@etri.re.kr (H.S.K.); hbcho@etri.re.kr (H.B.C.)

**Keywords:** ITS (Intelligent Traffic Systems), smart roadside server, infrastructure-based sensor, vehicle sensor

## Abstract

The use of newly emerging sensor technologies in traditional roadway systems can provide real-time traffic services to drivers through Telematics and Intelligent Transport Systems (ITSs). This paper introduces a smart roadside system that utilizes various sensors for driver assistance and traffic safety warnings. This paper shows two road application models for a smart roadside system and sensors: a red-light violation warning system for signalized intersections, and a speed advisory system for highways. Evaluation results for the two services are then shown using a micro-simulation method. In the given real-time applications for drivers, the framework and certain algorithms produce a very efficient solution with respect to the roadway type features and sensor type use.

## 1. Introduction

Telematics are defined as in-vehicle systems that offer active safety and infotainment services as well as location and traffic information via wireless communication technologies. In many countries, a variety of in-vehicle Telematics systems and Intelligent Transport Systems (ITS) are already available, while new systems are being currently designed.

According to plans from the U.S. Department of Transportation, the so-called ITS Strategic Research Plan (2010–2014), IntelliDrive projects dealing with ICT and transportation convergence technologies, including vehicle-to-vehicle (V2V) interaction and vehicle-to-infrastructure (V2I) interaction, are concurrently underway. One of the important items of the ITS Strategic Research Plan is making an intelligent roadside system under a Smart Roadside project, which began in 2008. The vision of a Smart Roadside is a system in which commercial vehicles, motor carriers, enforcement resources, highway facilities, intermodal facilities, toll facilities, and other transportation system nodes collect data for their own purposes. The nodes can then share the data seamlessly with relevant parties in order to improve motor carrier safety, security, operational efficiency, and freight mobility. In Europe, Cooperative Vehicle-Infrastructure Systems (CVIS) projects have been underway since 2006, and are supported by a grant from the European Commission. A precondition of the CVIS projects is that users of cooperative systems be made aware of these systems and understand how they can help drivers operate more safely, economically, and comfortably. Operators should also run their roadway networks in more cost- and eco-effective ways. Here, “cooperative” indicates systems that cooperate on two levels: (1) direct communication and the exchange of valuable information between entities; and (2) dynamic interaction amongst roadway users and transport infrastructure, which can provide win-win benefits for both drivers and roadway operators. The main elements of a high-level CVIS system are the vehicle, roadside system, and central system. In particular, a road side system includes standardized access, secure communication between vehicles and infrastructure, authentication, authorization, updates, and software configuration. Driven by the Korean government, the SMART highway R&D project was recently launched in 2008, and is expected to be completed by 2015. A SMART highway is a future high-speed roadway that supports an intelligent and convenient driving environment by providing roadway, vehicle, environmental, and human information, enabling users to concentrate solely on their driving and helping reduce accident rates. The SMART highway system uses a local server as a smart roadside server. This local server has some important functions. Through the IntelliDrive, CVIS, and SMART highway projects, researches regarding infrastructure-based sensors and OBD II-based vehicle sensors for safe driving have been ongoing. These infrastructure, in-vehicle, and smart roadside server sensors can enhance driver capability and perception.

In this research, we deal with Telematics/ITS service based on new IT technologies using a smart roadside server on a smart road, and we focus in particular on the system architecture and components, including service processing algorithm and issues. We focus particularly on two road safety situations: signalized intersections and the highway system. The problems relating to distributed roadside servers are more complicated than in internal distributed servers due to the complexity of a road environment. Bad weather such as snow, rain, and fog, and unstable traffic elements such as traffic jams, traffic accidents, pedestrians, and bicycles, can be considered at smart roadside servers. Other issues of consideration involve wireless communications and positioning systems, such as stable communication distance in Dedicated Short Range Communication (DSRC) devices, and Global Positioning System (GPS) errors.

The remainder of this paper is organized as follows: Section 2 introduces sensor technologies such as vehicle sensors, infrastructure sensors, and smart roadside server frameworks. In Sections 3 and 4, we suggest a system model for a roadside server, along with a processing algorithm, evaluation results based on simulation tools, and methods for driver assistance/safety alarms. Finally, in Section 5, we offer some concluding remarks regarding the research described in this paper, and provide some perspectives on future work.

## 2. Infrastructure Based Sensor and OBDII Based Vehicle Sensor

### 2.1. Road System and ITS Technology

**Figure 1.**
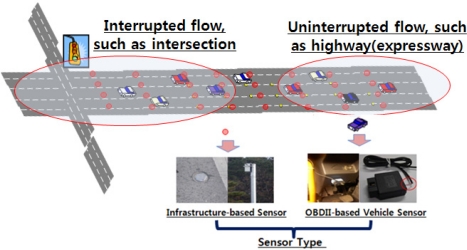
Roadway system and ITS technology.

The goals of ITS strategies are reducing travel time, easing delays and congestion, improving safety, and reducing pollutant emissions without the need for new roadway construction. ITS strategies that contain electronic surveillance, communications, and traffic analysis and control technologies bring about benefits to transportation system users and managers. ITS sensors often serve as data-gathering elements of an ITS, and therefore dictate ITS operating characteristics, types of data provided, and installation requirements. In particular, ITS and Telematics departments have gradually increased their interest in vehicle data through access to industry-standardized in-vehicle interfaces. This attempt to gather vehicular information and access can be determined differently depending on the type of roadway and collecting sensors used in the service. As shown in Figure 1, roadway types are classified into two categories of flow: uninterrupted and interrupted. Uninterrupted flow facilities have no fixed elements, such as traffic signals, that are external to the traffic stream and may interrupt the traffic flow. Traffic flow conditions result from interactions among vehicles in a traffic stream, and between vehicles and the geometric and environmental characteristics of the roadway. Interrupted flow facilities have fixed elements, such as traffic signals, stop and yield signs, and other types of controls, which may interrupt traffic flow. To capture vehicle movement in real-time, a road sensor is required at each type of facility. There are two popular categories of sensors: infrastructure-based sensors and OBDII-based vehicle sensors. Infrastructure-based sensors include pressure detectors, inductive loop detectors, magnetic detectors, ultrasonic detectors, microwave detectors, infrared detectors, and image detectors. These types of sensors utilize a part of the signal control and traffic operation in an ITS. In contrast, vehicle sensors include GPS, automatic vehicle identification (AVI) using radio frequency identification (RFID) tags, and on-board diagnostics II (OBD)-based vehicle sensors, which are connected to an in-vehicle network.

### 2.2. Infrastructure-Based Sensor

**Figure 2.**
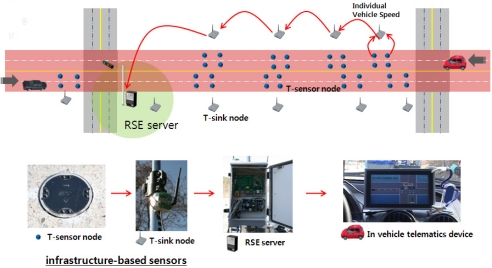
Examples of infrastructure-based sensors and procedures at an intersection.

In general, infrastructure-based sensors have certain strengths and weaknesses depending on their features, operation, and installation types. However, due to their cost-effectiveness in installation and operation, recent trends in other IT technologies include special traffic sensors for traffic detection. For example, sensors in the form of T-sensor nodes are randomly deployed around a target area where approaching lanes cross at an intersection. In other words, T-sensor nodes can acquire surrounding data, which they transmit to a roadside server through their neighbors based on a predetermined automatic mechanism; the user can then access a database to create a new service. As shown in Figure 2, these types of sensors consist of in-lane T-sensor nodes, T-sink nodes at roadsides, and local roadside equipment (RSE) servers. An RSE server located at the center of a crossroad gathers vehicular information from sensor nodes and transmits the gathered data to approaching vehicles. Using this collection and processing procedure, each vehicle that approaches the crossroad can be provided a new real-time service for avoiding potential traffic accidents.

### 2.3. OBDII-Based Vehicle Sensor

An OBDII-based vehicle sensor is a device within a vehicle that senses the conditions inside the vehicle every second. Data from a vehicle sensor include the speed, revolutions per minute (RPM), battery voltage, coolant temperature, coordinates, direction, distance travelled, Diagnostic Trouble Codes (DTC), and fuel consumption, which are supplied by the controller area network (CAN) bus. Through a vehicle sensor placed in each vehicle, vehicle sensor data can be formatted as probe data or messages, which are processed, formatted, and transmitted to a smart road server for further processing to create a clear understanding of the driving environment.

**Figure 3.**
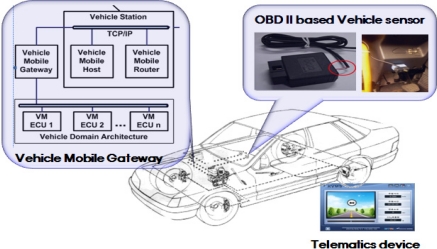
OBDII-based vehicle sensor.

As shown in Figure 3, an OBD II-based vehicle sensor is connected to the vehicle domain architecture designed by the vehicle manufacturer via a any gateway. The gateway is an interface device placed in a vehicle that acts as a mobile gateway between the vehicle's engine control unit (ECU) and an external device [ISO/DTR 13185-1.3].

### 2.4. Framework of Smart Road Server and Sensor System

**Figure 4.**
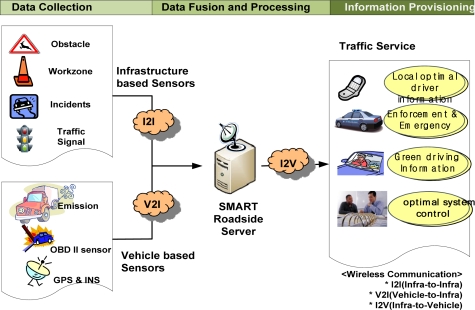
Framework of smart road server and sensor system.

The framework of a smart road server and sensor system is shown in Figure 4. The framework consists of five parts: infrastructure-based sensors, vehicle sensors, smart roadside server, wireless communication, and traffic service providers. A series of processes for a road and vehicle system consists of data collection, data fusion and processing, and information provisioning. In data collection, not only do infrastructure-based sensors collect certain obstacle data, work zone data, incidents, and traffic signal times, but vehicle sensors also collect emission data as well as vehicle speed, location, and RPM. These data can be transmitted to a smart roadside server using infrastructure-to-infrastructure (I2I) and V2I communication networks such as DSRC and Wireless Access for Vehicular Environments (WAVE). At a roadside server, these data undergo appropriate processing such as data fusion. The optimal information is then provided to drivers through a Telematics The framework of a smart road server and sensor system should have the following capabilities and requirements:

- Real-time context-aware computing and event stream processing
- Interoperability of various road sensors and vehicle data sensors
- Wireless and seamless communication (V2I) such as DSRC, WAVE, or Wi-Fi
- Distributed data sharing and transmitting to center server
- Optimal database design and policy
- Connection to Telematics and mobile phones
- Authentication, authorization
- Open standard platform

In particular, due to increasing user requests for real-time processing of large amounts of data coming from various data sources, the data processing paradigm has shifted from stored, static, and offline storage to streamed, dynamic, and online data sources. Some studies have been conducted on stream data processing like as vehicle and driver management system pattern query (VDMS-PQ) for the vehicle Telematics environment. Consequently, the smart road server and sensor system should be equipped adequate event stream processing method.

## 3. Application 1: Red-Light Violation Warning System at a Signalized Intersection

### 3.1. System Model

Several dangerous situations associated with high-speed approaches require special considerations when setting the signal timing intervals at a signalized intersection. For example, it may be difficult for a driver to decide whether to stop when the vehicle approaches a yellow light. The portion of the roadway before the intersection, where the driver may be indecisive (in stopping or proceeding into and through the intersection at the onset of a yellow light) is called the dilemma zone. Some drivers may violate a red signal due to this indecisiveness.

**Figure 5.**
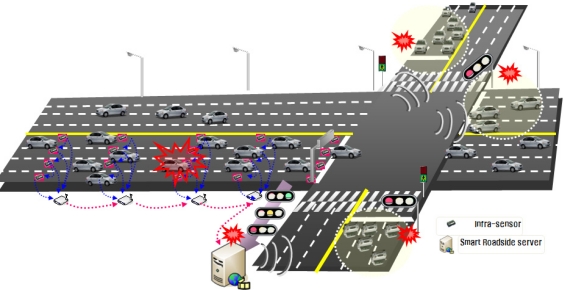
Red-light violation warning system at a signalized intersection.

As shown in Figure 5, as a vehicle approaches an intersection, we can decide whether it is in a dangerous position in the dilemma zone through the relationship between the remaining green or yellow times and the distance to the stop line. Real-time traveling data is collected at this service intersection through infrastructure-based sensors and vehicle sensors. A smart road server then processes a red-light violation warning algorithm, and the driver is provided the relevant information through a Telematics device.

In this research, we developed a red-light violation warning algorithm using the minimum safe stopping distance (SSD) and critical crossing distance. There is a relationship between the minimum SSD during the remaining time of a yellow signal and the critical safe crossing distance (CCD). A dilemma exists if SSD is greater than CCD for a vehicle upon data collection. If the intersection has sensor systems and a roadside server, each vehicle that approaches the crossroad can then predict and avoid possible traffic accidents.

### 3.2. Processing Algorithm

In this study, we developed a red-violation warning model for potential traffic conflicts at intersections based on real-time traffic data collected on vehicles in the approaching lane. This research also addresses the concept of potential traffic conflicts at an intersection. As a red-light violation warning model for signalized intersections, this research includes a red-light violation warning prediction model that considers the signal phase and remaining signal time when the vehicle’s location, speed, and time data are collected using multiple traffic sensors placed at the approaching lane.

**Figure 6.**
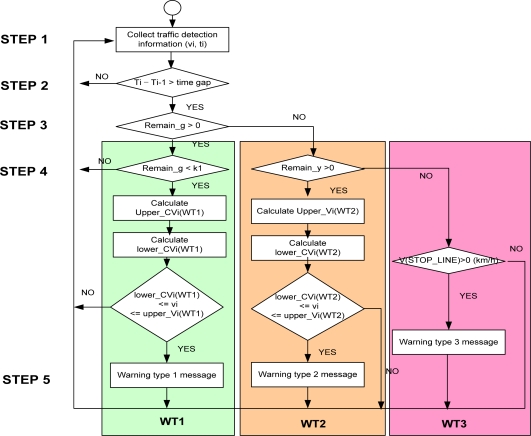
Flowchart for the red-light violation warning model.

A flowchart for the algorithm used in the model is shown in Figure 6. There are three types of wating time (WT), WT1, WT2 and WT3, provided in step 4.

- Step 1: Collect real-time vehicle speed and location data at an intersection using road sensors and vehicular data through V2I communication networks.
- Step 2: Calculate whether the time gap between continuous vehicles is longer than the critical length. If so, then go to step 3.
- Step 3: Calculate whether the remaining time for a green light is more than 0.
  If so, go to the WT1 process in step 4.
  Otherwise, go to the WT2 process in step 4.
- Step 4: In the WT1 case, if the remaining time for a green light is less than k1, calculate the red-light violation warning information as in Equations (1) and (2):
  (1) $$\mathrm{lower}\_{\mathrm{CV}}_{i}^{\mathrm{WT}1}=\frac{-t_{\mathrm{PRT}}+\sqrt{t_{\mathrm{PRT}}^{2}+\frac{2\cdot X_{i}}{d+G\cdot g}}}{1/(d+G\cdot g)}$$
  (2) $$\mathrm{upper}\_{\mathrm{CV}}_{i}^{\mathrm{WT}1}=\frac{Xi+(W+L)-\frac{1}{2}a{\left(remain\_g+extend\_y-t_{\mathrm{PRT}}\right)}^{2}}{\mathrm{remain}\_g+extent\_y}$$

In this case, if $\mathrm{lower}\_CV_{i}^{\mathrm{WT}1}\leq v_{i}\leq \mathrm{upper}\_{\mathrm{CV}}_{i}^{\mathrm{WT}1}$, a red-light violation is determined. The WT2 case calculates whether the remaining time for a yellow light is greater than 0. If so, the following equation of a dangerous red-light violation is calculated. Otherwise, proceed to the WT3 stage:

(3) $$\mathrm{lower}\_{\mathrm{CV}}_{i}^{\mathrm{WT}2}=\frac{-(t_{\mathrm{PRT}}+remain\_y-Y)+\sqrt{{\left(t_{\mathrm{PRT}}+\mathrm{remain}\_y-Y\right)}^{2}+\frac{2\cdot X_{i}}{d+G\cdot g}}}{1/(d+G\cdot g)}$$

(4) $$\mathrm{upper}\_{\mathrm{CV}}_{i}^{\mathrm{WT}2}=\frac{X_{i}+(W+L)-\frac{1}{2}a{\left(\mathrm{remain}\_y-t_{\mathrm{PRT}}\right)}^{2}}{\mathrm{remain}\_y}$$

In this case, if $\mathrm{lower}\_CV_{i}^{\mathrm{WT}2}\leq v_{i}\leq \mathrm{upper}\_CV_{i}^{\mathrm{WT}2}$, a red-light violation is determined. Finally, in the WT3 case, if the speed indicated at the stop line where the detecting sensor nodes are placed is more than 0 km/h during a red light, the potential for a collision is determined. Our proposed algorithms include the following variables and parameters:

– *v_i_* : vehicle speed indicated by a point detection sensor node at location i
– *t_PRT_* : driver’s perception and response time (s),
– *a* : acceleration rate (m/s^2^)
– *W* : intersection width (m)
– *L* : vehicle length (m)
– *d* : stationary deceleration rate (gravity rate × friction factor) (m/s^2^)
– *g* : gravity constant (9.8 m/s^2^)
– *G* : grade (m/m, %)
– *extend* _*y* : extended time (s)
– *remain* _*g* : remaining green time (s)
– *remain* _*y* : remaining yellow time (s)
– *X_i_* : distance between location i to stop line (m)
– $\mathrm{upper}\_{\mathrm{CV}}_{i}^{\mathrm{WT}1}$: critical speed at which a vehicle cannot stop in a WT1 case (km/h, m/s)
– $\mathrm{upper}\_{\mathrm{CV}}_{i}^{\mathrm{WT}2}$: critical speed at which a vehicle cannot stop in a WT2 case (km/h, m/s)
– $\mathrm{lower}\_{\mathrm{CV}}_{i}^{\mathrm{WT}1}$: critical speed at which a vehicle can stop in a WT1 case (km/h, m/s)
– $\mathrm{lower}\_{\mathrm{CV}}_{i}^{\mathrm{WT}2}$: critical speed at which a vehicle can stop in a WT2 case (km/h, m/s)

- Step 5: Provide a driver safety warning through a Telematics device using a V2I communication network

### 3.3. Evaluation Results Based on Microscopic Traffic Simulation

An evaluation of in- and out-of-vehicle traveller information systems is well-suited to virtual testing, which has an advantage over roadway studies, because it allows an examination of parameters of interest in difficult and critical driving situations without subjecting drivers to unnecessary risks. The first step in a simulation experiment is to design a simulated virtual intersection system that can replicate real-world traffic conditions and IT technology. To ensure the reliability and quality of the simulated results, this research has calibrated a simulation program. Table 1 summarizes the simulated conditions, such as traffic and road situations, signal control factors, and sensing points. After simulating using VISSIM scenarios, we analysed the *.mer file of each point sensor. Using algorithm-equipped automated software, we then performed the warning collision model using real-time synchronized signal times in Java-implemented software.

The scenarios used for the simulation testing are as follows: there were a total of 120 scenarios used based on roadway design speed, input volume, and driver perception response time parameters.

- Road design parameters: 50 km/h, 60 km/h, 70 km/h, 80 km/h (4 cases)
- Input volume parameters: 100–1,500 (vehicles/h) (15 cases)
- Driver perception and response times (*t_PRT_*): 1.5 s, 2 s (2 cases)

There are two main methods of verification for the developed model. One method compares the results from the red-light violation warning system with the field value from the ratio of red-light violations in real-world vehicular data The other method compares the results of simulation with the values from the simulation’s red-light violation ratio under simulation environments. We use the second method in which we locate the verification sensors within a dilemma zone at an intersection and analyze whether the vehicle collisions are forecasted. In other words, we compare the ID of a predicted red-light violation warning vehicle with the ID of a simulated vehicle in the red-violation warning system under a potential collision situation. Table 2 shows the verification results of the red-light violation warning system based on VISSIM tools. For verification, we use a remaining yellow light period of 1 s and a beginning red light period of 5 s. The results are the average values and percentages of all scenarios. In these results, there are 30.4 red-light violating vehicles and 524.4 non red-light violating vehicles predicted by the red-violation warning algorithm at simulation scenarios. In this case, there are around 25.3 really red-light violating vehicles in 30.4 red-light violating vehicles based on the vehicle ID verification. Thus, the percentage of red-light violation warning prediction is 88.5%, that is, 25.3 vehicles divided by 28.6 vehicles. Also, the correct classification rate is 98.5% (4.6% + 93.9%), where correct classification indicates whether red-light violating vehicles are properly classified. Thus, this current model is considered to have a very significant level of accuracy.

### 3.4. Driver Assistance and Safety Warning using In-Vehicle Telematics Device

**Figure 7.**
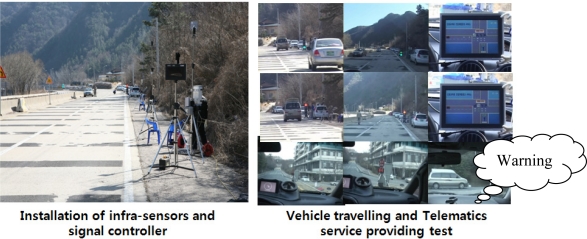
Signalized intersection test: red-light violation warning at signalized intersection.

If the discrimination results of red-light violation warning information indicate a red-light violation, a warning is provided to the vehicle’s Telematics device using a wireless communication network such as DSRC, WAVE, or Wi-Fi. Most fatal accidents at signalized intersections are caused by problems within the dilemma zone. The portion of roadway in advance of an intersection where a driver may be indecisive about stopping at the onset of a yellow light is called the dilemma zone. This method of driver assistance and safety warning can reduce the number of dilemma zone accidents. That is, the smart roadside sever system allows a driver to reduce his speed by notifying him when an impending collision is expected. With this information, the driver can safely stop at the stop line. Moreover, in cases in which a car is predicted not to stop at a stop line, the system provides information to the traffic signal controller, which creates a red traffic signal for all lanes. This research considers the possibility that the application of such a new service is possible for the safety of signalized intersections. In this case, the red-light violation warning model may be directly provided to all vehicle users. Figure 7 shows the simulation field test results about virtual signalized intersection in which the terminal in vehicles can be provided the red violation warning information to drivers.

## 4. Application 2: Advisory Speed Providing System on a Highway

### 4.1. System Model

**Figure 8.**
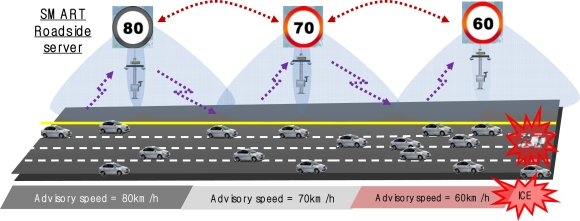
Advisory speed provisioning in a highway system.

A highway traffic system requires basic data such as vehicular data (speed, location), traffic flow, traffic control, safety information, road conditions, and weather information. A smart roadside system receives road surface data from each zone in a service area from at least one road sensor located in such a way as to calculate the road safety coefficient. The system also collects vehicular data from OBDII-based vehicle sensors in order to calculate the traffic flow coefficient. The system provides a vehicular safety service to a telematics device using the road safety coefficient of each zone, along with the traffic flow analysis coefficient. As shown in Figure 8, the recommend speed for an icy road is 60 km/h, which is calculated using traffic data and road surface sensing data. The approaching speeds are also reduced from 80 km/h to 70 km/h, before reaching the 60 km/h recommendation. These approaching speeds are delivered to the drivers.

### 4.2. Processing Algorithm

This system also provides driver assistance information through a Telematics device. The additional information includes advisory safety speed information considering road surface data such as snow, rain, obstacles, or ice. The advisory safety speed algorithm is equipped with a smart roadside server in the service area. The method for providing vehicular safety service includes receiving the road surface state data of each zone in the service area from infrastructure-based sensors; receiving location and running data from OBDII-based vehicle sensors; estimating the advisory safety speed based on the road surface status, location, and running data; and transmitting safe-distance risk information based on the advisory safety speed to the in-vehicle Telematics device.

Our advisory safety speed model consists of two parts: The first part is a macro car-following model that is used for considering highway traffic parameters, while the other is a safe stopping distance model for monitoring road-surface data. The formula used for advisory safety speed adv_u_j_ at time period *k* on road section *j* is shown in Equation (5). This algorithm is equipped at and performed by the smart roadside sever on a highway:

(5) $${\text{adv}}_{u_{j}(k)}=[t_{\mathrm{PRT}}+\sqrt{t_{\mathrm{PRT}}{}^{2}+\frac{2\cdot (t_{\mathrm{PRT}}\cdot u_{j}(k)+L)\cdot t_{\mathrm{PRT}}}{g(f_{j}(k)+G_{j}(k))}}]\cdot [g(f_{j}(k)+G_{j}(k))]$$

where:

- *t_PRT_*: driver perception and response time (s),
- *u_j_(k)*: the mean speed (or 85% of the top speed) at time period k on road section j (m/s),
- L: the spacing at a vehicle stop (vehicle distance + gap between vehicles),
- *f_j_(k)*: the road-friction parameter at time period k on road section j,
- *G_j_(k)*: the grade at link j during collection period k (%),
- *g*: the acceleration rate of gravity, 9.8 (m/s),
- *k*: collection time period k (s), and
- *j*: the number of road sections.

### 4.3. Evaluation Results Based on Microscopic Traffic Simulation

In this research, we also evaluated the advisory speed model using VISSIM. After simulating 12 different scenarios, as shown in Table 3, we then created the advisory safety speed model using a program in C++. The configuration of the study network used in the simulation is as follows:

- Length of road section = 1 km
- Number of lanes = 2
- Vehicle Capacity = 2,200 pcphpl (passenger car per hour per lane)
- Design speed = 110 km/h
- Location of events = 650–700 m from inflow point

The default parameters upon implementation of the simulation are as given below. Also, the algorithm update and information provisioning period is fixed at 5 min:

- *t_PRT_*: 2 s
- *G_j_(k)*: 0%
- L: 10 m

**Figure 9.**
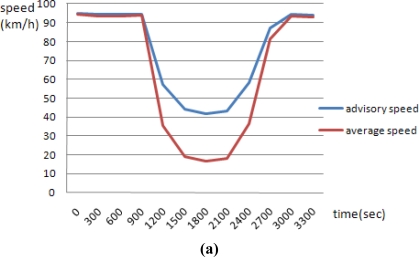

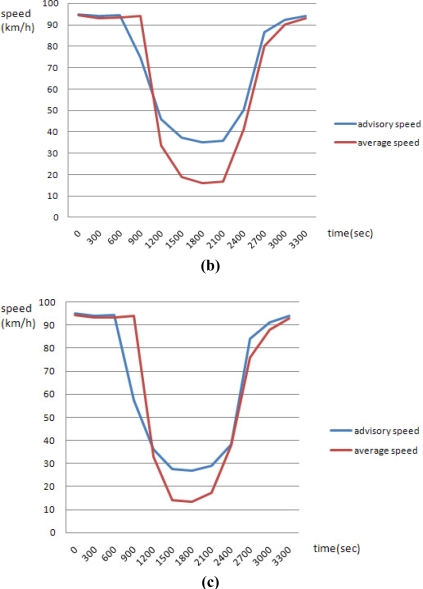
Simulation results. (**a**) Freeway-accident01 (Good traffic condition-accident-dry); (**b**) Freeway-accident11 (Good traffic condition-accident-wet); (**c**) Freeway-accident21 (Good traffic condition-accident-ice).

The simulation results for an advisory safety speed show that the most appropriate safety speed has a smaller stopping space than the mean speed for travelling vehicles. In Figure 9, the red line is the average speed per segment based on the simulation results, and the blue line is the advisory safety speed based on the suggested algorithm. For traffic simulation at the microscopic level, main parameters such as traffic condition [good/capacity (jam)], accident state (with/without accident), and road surface condition (dry/wet/ice) determine the advisory safety level and average speed. The results show that the advisory safety speed tends to decrease as the average speed decreases. In particular, the rate of decrease is quite sensitive in cases of accidents. When the traffic condition is at capacity, the average advisory safety speed is 60 km/h. This is 10 km/h less than under good conditions.

- Horizontal axis: time line (unit: s)
- Vertical axis: speed (unit: km/h)
- Red line: the average speed per segment based on the simulation results
- Blue line: the advisory safety speed based on the suggested algorithm

When the road surface is dry, wet, or icy, the average advisory safety speeds are 80 km/h, 76 km/h, and 68 km/h, respectively. Under an accident situation, the average advisory safety speed is 40 km/h, which is 40 km/h less than when no accidents have occurred. Figure 9(a–c) shows some of the simulation results for 12 scenarios under good traffic conditions and accident states, but where the state of the road surface state varies.

### 4.4. Driver Assistance and Safety Warning by an In-Vehicle Telematics Device

**Figure 10.**
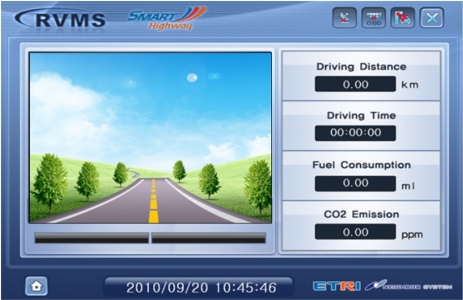
Example of a Telematics device with normal information.

**Figure 11.**
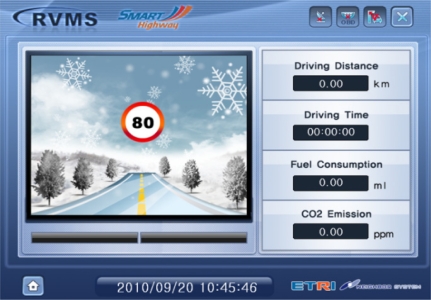
Example of a Telematics device with advisory speed and ice surface information.

As the results of the algorithm are advisory safety speeds for drivers, advisory safety information should be provided to in-vehicle Telematics devices using I2V. Figures 10 and 11 show examples of a GUI used in a Telematics terminal. As shown in Figure 11, the ‘80’ in the left-circle indicates the optimal advisory speed for road surface data such as ice, under the current traffic condition. This GUI displays the results from the developed Telematics device, and shows the total driving distance, total driving time, amount of fuel consumption, and CO_2_ emissions of the vehicle via an OBDII interface from an ECU. This device is very useful for driver assistance. In recent research, providing road surface warning signs has shown to be effective in reducing accident frequency and severity. Also, speed is not only related to the risk of being involved in a crash in the first place, but also affects the severity of a crash when one occurs. This processing method from a smart roadside server can be used as a new service model for uninterrupted roads that have implemented new information and communications technologies such as an Intelligent Vehicle Highway System (IVHS).

## 5. Conclusions

This paper has presented a framework for a smart roadside system and sensors such as infrastructure-base sensors and OBDII-based vehicle sensors. As application cases for this system, new real-time road services for two road types, signalized intersections and highway roads, were developed and applied to service algorithms for driver assistance and safety information services. The main services for the two road types are a red-light violation warning system at signalized intersections and an advisory speed provisioning system on highways. In this paper, the evaluation results of the simulation method are shown and applied to a real road and vehicle system for driver assistance and safety warnings.

Under the given real-time application for drivers, the framework and certain algorithms produce a very efficient solution with respect to roadway type features and sensor type use. As shown in some examples using an in-vehicle Telematics device, these technologies are very useful for a real-time application model in the present roadway system. This method was tested in a couple of vehicles at real intersections and highways. However, many vehicle and road types were not applied or tested through the micro traffic simulation tools. As a limitation of this study, the simulation method has certain errors related to the tool used, and has not been calibrated with real vehicle movements and roadway features. However, the suggested method can be applied for all vehicles equipped with Telematics devices and vehicle sensors, when roadway facilities have reliably collected vehicle and road data in real-time through infrastructure-based sensors. In reality, this service may be used in future roadway systems. After the installation and operation of various road and vehicle sensor systems, the qualitative and quantitative effects and benefits of this system can be demonstrated. Although this research is limited to certain sectors, the results of this paper demonstrate the feasibility of applying this new service system using infrastructure-based sensors and OBDII-based vehicle sensors. Under the given system applications, a framework utilizing certain algorithms will be a very efficient solution with respect to roadway-type features and sensor-type use.

## Figures and Tables

**Table 1.** Simulation input data.

**Categories**	**Detailed Description**	
Traffic conditions	Input volume (vehicles/h)	100, 200, 300, 400, 500, 600, 700, 800, 900, 1,000, 1,100, 1,200, 1,300, 1,400, 1,500 (Total of 15 cases)
	Turning rates	Left turn-through-Right turn: 15%–70%–15%
	Classification	Passenger cars: 90%, other vehicles: 10%
Road conditions	Number of lanes	4 lanes × 4 lanes
	Lane width	3.5 m
	Operation of lanes	Lane 1: left turn only, lanes 2–3: through only, lane 4: right turn only
Signal control	Cycle: 120 s, Green time in each direction: 27 s, Yellow time: 3 s, Red time: 90 s, Phase: Simultaneously with Left + Through direction	
Sensing point	A total of 50 point sensors in lanes 2 and 3. First location is the stop line, with 5 m intervals for further sensors	

**Table 2.** Verification of the model results.

**Classification**		**Real running vehicle status (no. of vehicles)(%) at simulation scenarios**		**Total no. of vehicles (%)**
		**Red-light violation vehicles**	**Non-red-light violation vehicles**	
Results of prediction (no. of vehicles in %) through suggested algorithms	Red-light violation vehicles	**25.3 (4.6%)**	5.1 (0.9%)	30.4 (5.5%)
	Non-red-light violation vehicles	3.3 (0.6%)	**521.1 (93.9%)**	524.4 (94.5%)
Total no. of vehicles (%)		**28.6 (5.1%)**	526.3 (94.9%)	554.9 (100%)

**Table 3.** Simulation scenarios.

**Traffic condition**	**Accident status**	**Road surface status (*f_j_* (*k*) )**	**Scenario ID**
LOS = C (Input Volume = 1,500 pcphpl)	without accident	Dry (f = 0.8)	Freeway-Basic01
		Wet (f = 0.4)	Freeway-Basic11
		Iced (f = 0.2)	Freeway-Basic21
	with accident (accident time = 1,200–2,400 s)	Dry (f = 0.8)	Freeway-accident01
		Wet (f = 0.4)	Freeway-accident11
		Iced (f = 0.2)	Freeway-acciden21
LOS = F (Input Volume = 2,300 pcphpl)	without accident	Dry (f = 0.8)	Freeway-Basic02
		Wet (f = 0.4)	Freeway-Basic12
		Iced (f = 0.2)	Freeway-Basic22
	with accident (accident time = 1,200–2,400 s)	Dry (f = 0.8)	Freeway-accident02
		Wet (f = 0.4)	Freeway-accident12
		Iced (f = 0.2)	Freeway-accident22
